# Spatio-temporal evolution of stroke mortality in Minas Gerais, Brazil, 1980-2021

**DOI:** 10.1590/S2237-96222024v33e20240017.en

**Published:** 2024-12-09

**Authors:** Daniel Hideki Bando, Francisco Chiaravalloti, Alfredo Pereira de Queiroz

**Affiliations:** 1 Universidade Federal de Alfenas, Instituto de Ciências da Natureza, Alfenas, MG, Brazil; 2 Universidade de São Paulo, Faculdade de Saúde Pública, São Paulo, SP, Brazil; 3 Universidade de São Paulo, Faculdade de Filosofia, Letras e Ciências Humanas, São Paulo, SP, Brazil

**Keywords:** Accidente Cerebrovascular, Registros de Mortalidad, Análisis Espacio-Temporal, Distribución Temporal, Estudios Ecológicos, Stroke, Mortality Registries, Temporal Distribution, Spatio-Temporal Analysis, Ecological Studies

## Abstract

**Objective:**

To analyze spatio-temporal evolution of stroke mortality in Minas Gerais state, Brazil, 1980-2021.

**Methods:**

Ecological study with aggregated data by micro-region. Segmented linear regression was used for trend analysis; maps with rates per five-year period and scan statistics were used for spatial analysis.

**Results:**

There were 392,521 stroke-related deaths (rate of 52.6/100,000-year). All rates (crude, adjusted, by age group) showed a decreasing trend, less so in the crude rate (Annual Percent Change [APC] = -0.70) and a faster decrease in the 20-39 age group (APC = -4.48). A high-rate cluster was identified in the southern region (1980-1999; Relative Risk [RR] = 2.06), and a low-rate cluster in the northwest (2008-2021; RR = 0.59). The most significant decrease occurred in the south (APC = -3.64).

**Conclusion:**

Stroke mortality showed a decreasing trend. Clusters and areas with higher rates identified in the northeast in recent years require attention by service managers.

## Study contributions

**Main results:**

Decreasing trend in stroke mortality, a cluster was found in the south with high rates (1980-1999; relative risk [RR] = 2.06) and another in the northwest with low rates (2008-2021; RR = 0.59).

**Implications for services:**

For health service managers, greater attention is suggested in the micro-regions and respective municipalities in the northeast that have presented a higher risk of mortality from stroke in recent years, with the aim of improving the regulation of health care service and network regulation, as well as optimizing access to these services.

**Perspectives:**

It is necessary to monitor the spatio-temporal evolution of stroke mortality and conduct new studies on different scales (for example, on the municipal scale) and methodologies (for example, multilevel analysis), to estimate risk factors and the social context of vulnerable populations.

## RESUMEN

**Objetivo:**

analizar la evolución espacio-temporal de la mortalidad por accidentes cerebrovasculares (ACV) en Minas Gerais, 1980-2021.

**Métodos:**

estudio ecológico, con datos por microrregión. En el análisis de tendencia se utilizó regresión lineal segmentada, en el análisis espacial estadística scan.

**Resultados:**

Hubo 392.521 muertes por ACV (tasa de 52,6/100.000-años). Todas las tasas (bruta, ajustada, grupos de edad) indicaron una tendencia decreciente, con menor velocidad en tasa bruta (Variación Porcentual Anual [VPA] = -0,70), mayor en el grupo de 20-39 años (VPA = -4,48). Se identificó un conglomerado con tasas altas en el sur (1980-1999; Riesgo Relativo [RR] = 2,06) y uno con tasas bajas en el noroeste (2008-2021; RR = 0,59). El descenso fue más intenso en el sur (VPA = -3,64).

**Conclusión:**

Se observó tendencia de disminución de mortalidad. La identificación de conglomerados y áreas con mayores tasas en el noreste en los últimos años merece la atención de los gestores.

## INTRODUCTION

Cardiovascular disease is the main cause of death in the world and in Brazil as well.^1^ The global age-adjusted stroke mortality rate in 2019 was 84.2/100,000 inhabitants; in Brazil it was 58.1/100,000 inhabitants, with significant regional variations.^1^ In Brazil the rate varied from a minimum of 44.2/100,000 inhabitants, in the state of Rio Grande do Norte, to a maximum of 91.6/100,000 in the state of Maranhão. The states in the North region had the highest rates. In the state of Minas Gerais, the rate was slightly below the national average (50.1/100,000 inhabitants), however, as in the rest of the country, there are intrastate variations.^1^ In Brazil, in 2022, the burden of mortality due to cardiovascular disease was 400,154 deaths (25.9% of the total). Of this total, 120,658 (30.2%) corresponded to ischemic heart diseases (for example, angina and heart attack) and 107,322 (26.8%) to strokes.^2^

Globally, from 1990 to 2019, the stroke mortality rate decreased significantly, with annual percentage change (APC) of -1.58, having decreased most intensely in high-income countries.^3^ From 1996 to 2015, Brazil also had a decreasing trend (APC = -2.4), with regional differences, and in the states of the Southern and Southeast regions the decrease was more pronounced. In the Midwest region, there was also a reduction, while in the Northern and Northeast regions there was a division: the trend increased in some states; was stable in others; and only decreased in a few.^4^

According to data from the 2019 National Health Survey (*Pesquisa Nacional de Saúde*), conducted by Brazilian Institute of Geography and Statistics (*Instituto Brasileiro de Geografia e Estatística* - IBGE), 3.1 million people aged 18 or over reported having had a stroke diagnosed. Stroke prevalence is directly related to age, varying from 0.3%, among people aged 18 to 29, to 9.5% for people aged 75 or over.^5^ According to the Ministry of Health, there were 107,322 deaths due to strokes in Brazil in 2022.^2^ These conditions significantly impact the Brazilian National Health System (*Sistema Único de Saúde* - SUS), in which 164,000 hospitalizations due to strokes were recorded in 2021, with annual expenditure estimated at BRL 250 million. In primary health care, in 2021, there were more than 102,000 stroke consultations, not counting rehabilitation care.^6^

In addition to the economic impact on health services and related to physical disability resulting from strokes, it is known that stroke mortality is related to people who have poorer socioeconomic conditions, such as low level of education and lower per capita income.^7,8^ As such, in the same way as infant mortality, stroke mortality is an important social indicator. Part of stroke incidence and mortality could be avoided by addressing behavioral risk factors, such as smoking, harmful use of alcohol, unhealthy diet, obesity and physical inactivity.^3^

Ecological epidemiological studies on the spatio-temporal evolution of stroke mortality are fundamental for the formulation of public policies, such as prevention, access to health services and health promotion.^9^ Furthermore, they can be replicated in different populations, as well as suggesting new hypotheses and serving to guide new studies. Given the scarcity of studies on this topic in the state of Minas Gerais, the objective of this article is to analyze the spatio-temporal evolution of stroke mortality rates in that state, from 1980 to 2021. 

## METHODS

**Figure 1:**
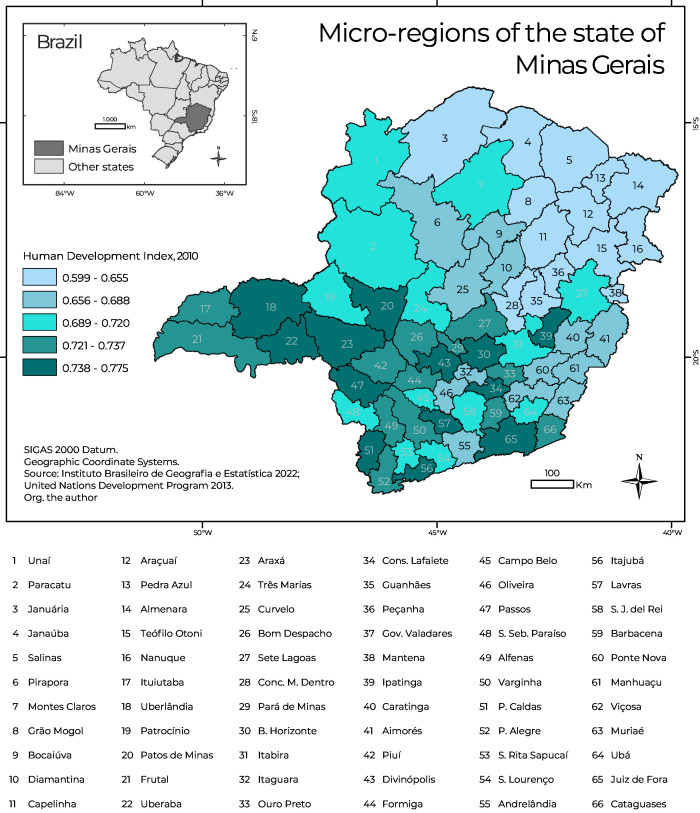
Map showing the location of the micro-regions of Minas Gerais, Brazil, with human development index

We conducted an ecological study on the spatio-temporal evolution of stroke mortality in the state of Minas Gerais, Brazil, for the period from 1980 to 2021. The units of analysis were the state’s microregions. The microregions were chosen because, of the 853 current municipalities in the state, 97 were only created in 1995. Therefore, the study using microregions made it possible to include a broad time series available since 1980. According to data from the 2022 Demographic Census, Minas Gerais had a population of 20.5 million inhabitants,^10^ spread over 66 microregions (Figure 1). The Belo Horizonte microregion was the most populous, with around 5.3 million inhabitants. 

The Human Development Index (HDI) is a synthetic indicator that assesses the level of development of a society, consisting of three dimensions: income, education and health. The Minas Gerais HDI, in 2021, was 0.774, the fourth highest index among the 27 Brazilian Federative Units.^10^ The spatial distribution of the HDI by microregions will only be used to describe the overall socioeconomic conditions of Minas Gerais (Figure 1). There is strong social inequality: the micro-regions in the *Triângulo* *Mineiro* region (Uberlândia, Uberaba), the metropolitan region (Belo Horizonte) and the south (Juiz de Fora, Lavras) presented HDIs above 0.738; however, in the northeastern portion of the state, Grão Mogol, Araçuaí, Peçanha and Almenara had HDIs below 0.611.^11^

All stroke deaths in Minas Gerais, in the period evaluated, were classified according to the following variables:

sex (male; female);
age (in years: ≤19, 20-39, 40-49, 50-59, 60-69, 70-79, ≥80);
place of residence of the death (micro-region);
year of death (1980–2021).

During the study period, of the total of 392,521 deaths, 441 (1.1%) did not have information on the micro-region of residence, therefore they were not used in the spatial analysis.

Mortality data were extracted from the Ministry of Health Mortality Information System (*Sistema de Informação sobre Mortalidade* - SIM),^2^ according to the micro-region of residence of the registered deaths. For the period from 1996 to 2021, deaths due to stroke correspond to codes I60 to I69 of the Tenth Revision of the International Statistical Classification of Diseases and Related Health Problems (ICD-10). For the period from 1980 to 1995, deaths due to stroke are covered by codes 430 to 438 of ICD-9.^2^ Population data by age group, sex and period were obtained from demographic censuses, with the respective projections for intercensal periods, available via the portal of the Information Technology Department of the Brazilian National Health System (DATASUS).^2^ The cartographic base of the study was made up of the territorial mesh of the state of Minas Gerais, by microregions, in shapefile format, available via the IBGE portal.^10^

We used the Joinpoint Regression Program, version 5.0.2.12, to analyze trends in mortality rates for the state of Minas Gerais. Crude, age-adjusted and age-specific mortality rates were calculated. The calculation of the crude mortality rate was based on the number of deaths due to strokes among residents in a given location, considered as the numerator, and the total resident population, as the denominator, multiplied by 100,000, in the period under consideration. The specific rate by age group took into account the number of deaths in an age group divided by the population in the same age group. The age-adjusted mortality rate was calculated using the direct method, taking the World Health Organization (WHO) standard population.^13^ This approach adjusts the crude rates according to the age distribution of an external, arbitrarily defined population (in this case, the WHO standard population), which is useful for comparing populations of different age structures.

The Joinpoint Regression Program uses the segmented log-linear Poisson regression model, which applies the Monte Carlo permutation test to identify points where the trend line changes significantly in magnitude or direction. The analysis starts with the minimum number of joinpoints (zero, which is a straight line), and tests whether one or more joinpoints are statistically significant and should be added to the model. The APCs and 95% confidence intervals (95%CI) were estimated for the time segments on either side of the joinpoints. For this study, the parameters selected were: grid search method (the joinpoints occur exactly in the observations), two minimum observations between joinpoints and a maximum of five joinpoints per analysis. The Joinpoint program has been used in the area of epidemiology to estimate mortality trends due to various causes, including stroke.^4,14-16^

Choropleth thematic maps^17^ were produced to describe the evolution of the spatial pattern of mortality rates. Rates adjusted by sex and age were used, grouped into five-year periods, with the exception of the last period (seven years): 1980-1984, 1985-1989, 1990-1994, 1995-1999, 2000-2004, 2005-2009, 2010-2014, and 2015-2021. The Jenks method was used to define the legend intervals.

Identification of spatio-temporal clusters was performed using scan statistics, with the SaTScan program version 10.1.3.18. The scan statistic inserts a circular window of variable size at the centroid of each area, so that the radius increases in size, sweeping and adding the centroids from neighboring areas. The size of the cluster, or spatial window, cannot exceed 50% of the population. Under the null hypothesis, the relative risk (RR) of the cluster is calculated and it is verified whether it occurred by chance, using the likelihood ratio test based on Monte Carlo simulations. In this analysis, the “age group” and “sex” variables were considered as adjustment variables, of the offset type. Four types of analysis were performed to identify clusters in SaTScan: i) Purely spatial, ii) spatio-temporal for high rates, iii) spatio-temporal for low rates and iv) spatial variation in temporal trends. SaTScan considers the cluster to have high rates when RR is significantly greater than 1, and low rates when RR is less than 1. The spatio-temporal analysis identifies in which period the RR of the respective clusters were significant. The temporal window cannot exceed 50% of the study period. The analysis of spatial variation in temporal trends identifies the APC of rates within and outside clusters. A 5% significance level was adopted for all tests. We used the ArcGIS program version 10.6 to create the maps.

In accordance with National Health Council Resolution No. 466/2012, as the study used public domain secondary data from DATASUS, it therefore did not need to be submitted for appraisal by a Research Ethics Committee. 

## RESULTS

**Figure 2:**
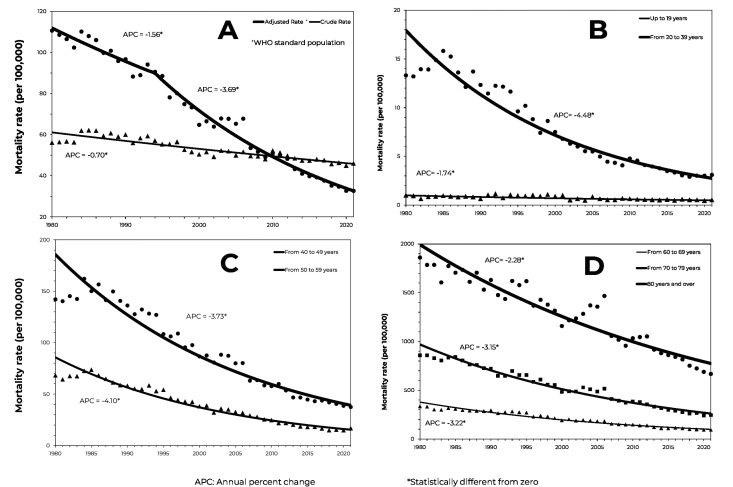
Trends in stroke mortality rates (per 100,000), in Minas Gerais, Brazil, 1980-2021:

During the period studied, there were 392,521 deaths due to strokes, which corresponds to a mortality rate of 52.6/100,000 inhabitants-year. Figure 2 shows trends in stroke mortality rates in Minas Gerais, from 1980 to 2021. The points represent the observed values, and the lines correspond to the trends in the regression analysis. Both the crude and adjusted rates showed a significant decreasing trend. The decrease was more rapid taking the adjusted rate, especially from 1994 onwards, with APC of -3.69 until the end of the period (Figure 2A).

The youngest age groups had the lowest mortality rates. The trend analysis revealed that all age groups showed a significant decreasing trend throughout the period, that is, zero joinpoint. The 20-39 age group showed the highest speed of decrease (APC = -4.48), while the age group up to 19 years old showed the slowest (APC = -1.74) (Figure 2B). 

**Figure 3:**
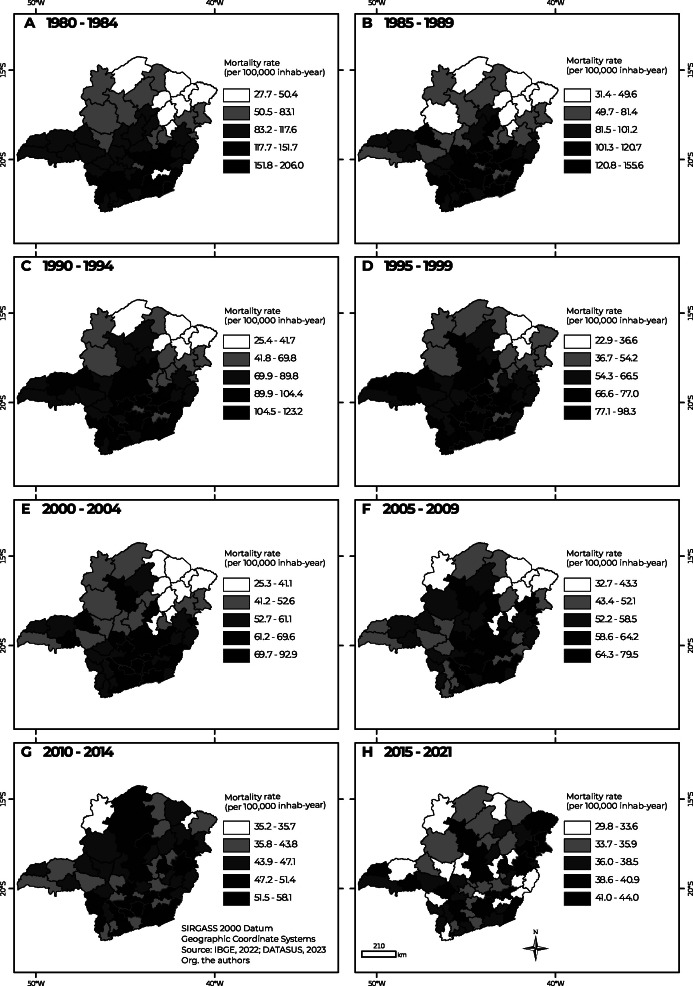
Stroke mortality rates (per 100,000) adjusted for sex and age, in Minas Gerais, Brazil, 1980-2021, by five-year periods (except the last period which is seven years)

Figure 3 shows the spatio-temporal evolution of stroke mortality rates. It can be seen that the first five-year periods had the greatest amplitudes of change in rates and also a reduction in rates throughout the period. In the first five-year period, the average rate was 98.6/100,000 inhabitants. Higher rates, above 150/100,000, can be seen in the southeast and southern portions of the state, in the Cataguases, Barbacena and Juiz de Fora microregions. On the other hand, Grão Mogol, Januária and Araçuaí, to the north, had rates lower than 35/100,000 inhabitants (Figure 3A).

In the second five-year period, the spatial pattern was similar (Figure 3B). The micro-regions of the metropolitan region also stood out, such as Belo Horizonte, Ouro Preto, Pará de Minas, Itabira, Conselheiro Lafaiete, with rates above the average. In the third and fourth five-year periods, the average rates were 85.2/100,000 inhabitants and 66.7/100,000 inhabitants, respectively. The spatial pattern remained, with higher rates in the south and southeast (Figure 3C and D). In the fifth and sixth five-year periods, the average rates were 57.7/100,000 inhabitants and 55.4/100,000 inhabitants, respectively (Figure 3E and F).

In the seventh five-year period, the state’s average rate was 46.8/100,000 inhabitants. A new pattern began to form, with high rates in microregions in the north (Bocaiúva) and east (Governador Valadares) (Figure 3F). In the last period of the series, the average rate was 36.8/100,000 inhabitants, the lowest in the entire period covered by the study (Figure 3H). The south lost its leading role; in the eastern portion, the micro-regions of Nanuque, Teófilo Otoni and Governador Valadares stood out, with high rates. 

**Figure 4:**
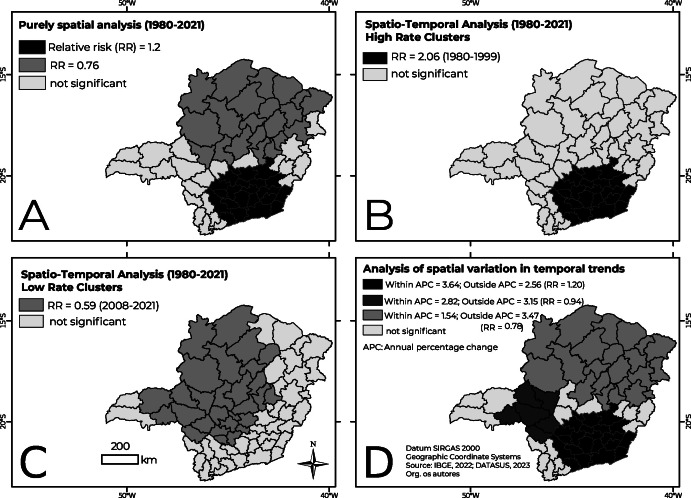
Spatial scan test of stroke mortality in the state of Minas Gerais, Brazil, 1980-2021: A) Purely spatial analysis, B) Spatio-temporal analysis for high rates, C) Spatio-temporal analysis for low rates, D) Analysis of spatial variation in temporal trends

Figure 4 shows the results of the spatial scan test. In the purely spatial analysis, two clusters were detected, one of which with high rates, in the south, composed of 22 areas, including 75% of the microregions in the south of the metropolitan region (Belo Horizonte, Conselheiro Lafaiete, Itabira, Itaguara, Ouro Preto, Pará de Minas). There the relative risk was 1.20; as such, residents in this cluster have a 20% greater risk of dying from a stroke compared to areas outside the cluster (Figure 4A). On the other hand, to the north a cluster of low rates was identified, formed by 21 areas. The risk of dying from a stroke in this area falls by 24% (Figure 4A). 

The spatio-temporal analysis for high rates identified a cluster in the period from 1980 to 1999 (Figure 4B), formed by the same 22 microregions as in the purely spatial analysis (Figure 4A). However, in this cluster, from 1980 to 1999, risk of death from stroke was twice as high. The spatio-temporal analysis for low rates identified a cluster in the period from 2008 to 2021, formed by 28 areas in the northwest and west of the state (Figure 4D). In this area, the risk of death from stroke falls by 41%. This cluster included the Belo Horizonte microregion and the western portion of the metropolitan region. Finally, the analysis of spatial variation in temporal trends revealed that the decreasing trend was greater in the high rate cluster (APC = -3.64) (Figure 4C).

## DISCUSSION

Stroke mortality in Minas Gerais has shown a downward trend in the crude rate, in the age-adjusted rate and in all age groups. A study evaluated trends in stroke mortality rates in the world, from 1990 to 2019, with data from the Global Burden of Disease project.^3^ The global rate of stroke deaths indicated a decreasing trend, with APC = -1.58. That study created a synthetic sociodemographic index, composed of the following variables: per capita income, average education and total fertility rate. The index was categorized into five quintiles: high, medium-high, medium, medium-low and low. Countries with a high sociodemographic index showed reduction at a faster rate (APC = -2.83), which gradually decreased, passing through the average index, which includes Brazil (APC = -1.37), to countries with a low sociodemographic index (APC = -1.21). 

Trends in stroke mortality rates were studied in Brazil, by sex and age group, from 2000 to 2018.^16^ That study described a decrease in all age groups, being more intense in the 35 to 44 age group, with a reduction of 52.1% in men and 53.2% in women. However, it did not report the APCs and their respective statistical significance, but rather presented the results in graphs. Minas Gerais appears to follow the national pattern, given that the speed of decrease was more intense in the 20-49 age groups.

Trends in stroke deaths were also analyzed in Brazil by state and region, from 1996 to 2015.^4^ For Brazil as a whole, APC was -2.4. All states in the Southern, Southeast and Midwest regions showed a decreasing trend in the period, which was more intense in the South and Southeast. In the Northeast, more than half of the municipalities showed an increasing trend. In the North, the trend was predominantly stable. Despite the decline in stroke mortality in Brazil, there was an increase in mortality due to hypertension, possibly related to the filling out of death certificates, a reduction in ill-defined causes and an increase in hypertension itself.^19^ Again, Minas Gerais appears to follow the national pattern, given that the rate of reduction in rates was greater in the southern portion of the state.

In part, this spatio-temporal pattern of stroke deaths can be explained by the epidemiological transition process. In Brazil, there was a delay of a few decades in this process in relation to high-income countries. In countries such as the United States, Canada and Western European nations, mortality due to cardiovascular disease began to decline in the late 1950s.^20^ The main change in the Brazilian pattern occurred in the 1960s, when mortality due to cardiovascular diseases surpassed infectious diseases.^21^ Mortality due to cardiovascular disease continued to grow, before beginning to decrease from the 1980s onwards.^20,22^ Furthermore, in Brazil, the transition occurred over a long period of time, with a slight reduction and reemergence of infectious diseases, such as dengue. Additionally, there are regional differences, with the North and Northeast lagging behind the South and Southeast.^20^

Several factors are related to the epidemiological transition process, such as access to health services,^20^ increased life expectancy and urbanization rate,^21^ improved sanitary and nutritional conditions, increased vaccination coverage and changes in habits, such as reducing smoking.^23,24^ In Minas Gerais, information on the cause of death by health macro-regions, collected by the SIM system,^2^ suggests prolonged transition and delay in the north in relation to the south. In the south, deaths due to cardiovascular diseases decreased from 33.1% in 1980 to 26.7% in 2019. In the northern macro-region, in the equivalent period, they increased from 14.9% to 21.6%. In the case of infectious diseases, in the southern macro-region, they decreased from 7.5% in 1980 to 2.5% in 2019. In the northern macro-region, they decreased from 11.4% to 6.2%.

In Brazil, studies on the spatio-temporal evolution of stroke mortality with intrastate information are scarce. The spatio-temporal evolution of stroke mortality was analyzed in the elderly population in the state of Alagoas, from 2000 to 2016, using trend analysis and the Moran statistic.^15^ The study identified an increasing trend until 2007 (APC = +3.9) followed by a decreasing trend until 2016 (APC = -1.4). As for the spatial pattern, it identified a high-high cluster to the east, including the capital Maceió, and a low-low cluster to the west of the state.^15^ The downward trend from 2007 onwards, as well as the risk area, which includes the capital, may be evidence of delay in the epidemiological transition process. 

The spatio-temporal evolution of stroke mortality was studied in the state of Rio de Janeiro, by aggregating municipalities into regions, with the exception of the capital and the city of Niterói,^25^ in three periods: 1979-1989, 1990-1999, and 2000-2010. In the first period, rates were higher in the mountainous and northwestern regions, and lower in the south, in municipalities that included the capital and Niterói. All regions showed a decrease in stroke mortality, with this being higher in the mountainous and northwestern regions. The capital and Niterói maintained low rates in subsequent periods, and in the third period, there was homogenization throughout the state. 

Other studies have investigated the spatio-temporal evolution of mortality due to cardiovascular diseases, which include stroke.^4,9,26,27^ A study conducted in the state of Ceará performed spatial analysis of mortality due to cardiovascular diseases, between 2009 and 2019, using Local Moran’s I.^9^ A high-high cluster was identified in the south of Ceará, and a low-low cluster on the north coast and in the Metropolitan Region of Fortaleza. In Ceará, a tendency for an increase in stroke mortality rates from 1996 to 2007 (APC = +2.8) and a decrease from 2007 to 2015 (APC = -2.2) was also identified.^4^ This information also suggests the delay in epidemiological transition in the Northeast region of Brazil. 

In the state of Paraná, a study investigated the spatial distribution of mortality due to cardiovascular diseases by health macro-regions, in the three-year periods 1989-1991 and 2006-2008. ^26^ In the first three-year period, the northern macro-region had the highest rate, while the west had the lowest. In the second three-year period, the northwest macro-region had the highest risk, while the east, which includes the capital, had the lowest risk. However, the study did not detect spatial dependence using Global Moran’s I. The spatial pattern of several causes of death, including cardiovascular diseases, were analyzed in the state of São Paulo, in 2000, 2008 and 2016.^27^ That study identified a greater concentration of deaths in the southwest and northwest in all three years, and in 2016 some hotspots were identified in the north of the state. 

Our study has some limitations. One of them is possible errors in filling out death certificates, such as possible errors related to ICD-10 and ICD-9, which may vary in different regions.^19,20^ It is known that there has been an improvement in the quality of information relating to the SIM system in recent decades. The proportion of deaths due to ill-defined causes (Chapter XVIII of ICD-10)^28^ in Minas Gerais, in 1996, was 14.4%, this being below the national average (15.1%) and below the Northeast region (32.4%), but above the Southeast region (9.2%). In turn, in 2021, there was an improvement, and the proportion decreased in general: Minas Gerais (5.9%), national average (5.1%), Southeast (5.7%) and Northeast (5.9%).^2^ Another limitation is inherent to the study design. We are referring to ecological fallacy, that is, attributing results found in population aggregates to the individual. The adopted unit of analysis, the microregion, can also hide inequalities at the municipal level. Data incompleteness (1.1%) did not affect the results.

Conclusively, the present study identified, in Minas Gerais, a general trend of reduction in stroke mortality. The identification of clusters, as well high rates being found in recent years in the northeast of the state, can help health service managers in formulating public policies to reduce risks and mortality due to strokes, promoting a better quality of life for the population.
