# Supplementary material for: Spatio-temporal evolution of stroke mortality in Minas Gerais, Brazil, 1980-2021
Source: Epidemiol Serv Saude. 2024 Dec 9;33:e20240017. doi: 10.1590/S2237-96222024v33e20240017.en (PMC11654715; doi:10.1590/S2237-96222024v33e20240017.en)
Supplement: Translation

# Translation: Evolução espaço-temporal da mortalidade por acidente vascular cerebral em Minas Gerais, 1980 a 2021

**DOI:** 10.1590/S2237-96222024v33e20240017.pt

**Authors:** Daniel Hideki Bando, Francisco Chiaravalloti, Alfredo Pereira de Queiroz

**Affiliations:** 1 Universidade Federal de Alfenas, Instituto de Ciências da Natureza, Alfenas, MG, Brasil; 2 Universidade de São Paulo, Faculdade de Saúde Pública, São Paulo, SP, Brasil; 3 Universidade de São Paulo, Faculdade de Filosofia, Letras e Ciências Humanas, São Paulo, SP, Brasil

**Keywords:** Acidente Vascular Cerebral, Registros de Mortalidade, Distribuição Temporal, Análise Espaço-Temporal, Estudos Ecológicos

## Abstract

**Objetivo:**

Analisar a evolução espaço-temporal da mortalidade por acidente vascular cerebral (AVC) em Minas Gerais, 1980 a 2021.

**Métodos:**

Estudo ecológico, com dados agregados por microrregião. Na análise de tendência, foi utilizada regressão linear segmentada; na análise espacial, mapas com taxas quinquenais e estatística *scan*.

**Resultados:**

Ocorreram 392.521 mortes por AVC (taxa de 52,6/100 mil habitantes-ano). Todas as taxas (bruta, ajustada, por faixas etárias) apresentaram tendência de diminuição, com velocidade menor na taxa bruta (variação percentual anual [VPA] = -0,70) e maior na faixa etária de 20 a 39 anos (VPA = -4,48). Foi identificado um conglomerado no sul com taxas altas (1980-1999; risco relativo [RR] = 2,06), e no noroeste, com taxas baixas (2008-2021; RR = 0,59). A diminuição foi mais intensa no sul (VPA = -3,64).

**Conclusão:**

Verificou-se tendência de diminuição da mortalidade por AVC. A identificação de conglomerados e áreas com maior risco no nordeste do estado, nos últimos anos, merece atenção dos gestores.

## Contribuições do estudo

**Principais resultados:**

Tendência de diminuição na mortalidade por acidente vascular cerebral (AVC), um conglomerado no sul com taxas altas (1980-1999; risco relativo [RR] = 2,06) e outro no noroeste com taxas baixas (2008-2021; RR = 0,59).

**Implicações para os serviços:**

Aos gestores, sugere-se maior atenção nas microrregiões e respectivos municípios do nordeste que apresentaram maior risco de mortalidade por AVC nos últimos anos, com o objetivo de aprimorar a regulação dos serviços e redes de atenção à saúde, bem como otimizar o acesso a esses serviços.

**Perspectivas:**

É preciso monitorar a evolução espaço-temporal da mortalidade por AVC e conduzir novos estudos em diferentes escalas (por exemplo, na escala municipal) e metodologias (por exemplo, análise multinível), para se estimarem os fatores de risco e o contexto social das populações vulneráveis.

## INTRODUÇÃO

A doença cardiovascular é a principal causa de morte no mundo e no Brasil.^1^ A taxa global de mortalidade por acidente vascular cerebral (AVC) ajustada por idade, em 2019, foi de 84,2/100 mil habitantes; no Brasil, foi de 58,1/100 mil habitantes, com significativas variações regionais.^1^ Em nosso país, a taxa variou da mínima de 44,2/100 mil habitantes, no Rio Grande do Norte, até a máxima de 91,6/100 mil no Maranhão. Os estados da região Norte apresentaram as maiores taxas. Em Minas Gerais, a taxa foi um pouco abaixo da média nacional (50,1/100 mil habitantes), no entanto, assim como no restante do país, há variações intraestaduais.^1^ No Brasil, em 2022, a carga da mortalidade por doença cardiovascular foi de 400.154 óbitos (25,9% do total). Desse total, 120.658 (30,2%) corresponderam às doenças isquêmicas do coração (por exemplo, angina e infarto) e 107.322 (26,8%) ao AVC.^2^

Globalmente, de 1990 a 2019, a taxa de mortalidade por AVC diminuiu significativamente, com variação percentual anual (VPA = -1,58), tendo sido a velocidade mais intensa nos países ricos.^3^ O Brasil, de 1996 a 2015, também apresentou tendência de diminuição (VPA = -2,4), com diferenças regionais, e os estados do Sul e Sudeste apresentaram maior velocidade de redução. No Centro-Oeste, também houve redução, ao passo que no Norte e no Nordeste houve uma divisão: alguns estados apresentaram aumento; outros, estabilidade; e em poucos houve diminuição.^4^

Segundo dados da Pesquisa Nacional de Saúde de 2019, do Instituto Brasileiro de Geografia e Estatística (IBGE), 3,1 milhões de pessoas de 18 anos ou mais referiram diagnóstico de AVC. A prevalência do AVC está diretamente relacionada com a idade, variando de 0,3%, entre as pessoas de 18 a 29 anos, até 9,5% para as pessoas com 75 anos ou mais de idade.^5^ De acordo com o Ministério da Saúde, ocorreram 107.322 óbitos por AVC no Brasil em 2022.^2^ Essas condições impactam significativamente o Sistema Único de Saúde (SUS), onde foram registradas 164 mil internações por AVC em 2021, com gasto anual estimado em R$ 250 milhões. Na atenção primária à saúde, em 2021, houve mais de 102 mil atendimentos de AVC, fora os atendimentos de reabilitação.^6^

Além do impacto econômico nos serviços de saúde e relacionados à incapacidade física decorrente do AVC, sabe-se que a mortalidade por AVC está relacionada com piores condições socioeconômicas da população, como baixo nível de escolaridade e menor renda *per capita*.^7,8^ Portanto, assim como a mortalidade infantil, a mortalidade por AVC é um importante indicador social. Parte da incidência e mortalidade por AVC poderia ser evitada, combatendo-se os fatores de risco comportamentais, como tabagismo, uso nocivo de álcool, dieta pouco saudável, obesidade e inatividade física.^3^

Estudos epidemiológicos ecológicos sobre a evolução espaço-temporal da mortalidade por AVC são fundamentais para a formulação de políticas públicas, como prevenção, acesso aos serviços de saúde, promoção da saúde.^9^ Além disso, eles podem ser replicados em diferentes populações, bem como sugerir novas hipóteses e direcionar novos estudos. Dada a escassez de estudos dessa temática no estado de Minas Gerais, o objetivo deste artigo é analisar a evolução espaço-temporal das taxas de mortalidade por AVC, de 1980 a 2021. 

## MÉTODOS

Realizou-se um estudo ecológico sobre a evolução espaço-temporal da mortalidade por AVC no estado de Minas Gerais, para o período de 1980 a 2021. As unidades de análise foram as microrregiões. Optou-se pelas microrregiões porque, dos 853 atuais municípios do estado, 97 foram emancipados em 1995. Portanto, o estudo com as microrregiões permitiu incluir uma série histórica ampla e disponível desde 1980. De acordo com dados do Censo Demográfico de 2022, Minas Gerais contava com uma população de 20,5 milhões de habitantes,^10^ agrupada em 66 microrregiões (Figura 1). A microrregião de Belo Horizonte era a mais populosa, com cerca de 5,3 milhões de habitantes. 

**Figura 1:** Mapa de localização das microrregiões de Minas Gerais, com índice de desenvolvimento humano

O Índice de Desenvolvimento Humano (IDH) é um indicador sintético que avalia o grau de desenvolvimento de uma sociedade, sendo composto por três dimensões: renda, educação e saúde. O IDH de Minas Gerais, em 2021, foi de 0,774, o quarto maior índice entre as 27 Unidades da Federação (UFs).^10^ A distribuição espacial do IDH por microrregiões será usada apenas para descrever as condições gerais socioeconômicas de Minas Gerais (Figura 1). Nota-se forte desigualdade social: as microrregiões na região do Triângulo Mineiro (Uberlândia, Uberaba), da região metropolitana (Belo Horizonte) e do sul (Juiz de Fora, Lavras) apresentaram IDH acima de 0,738; entretanto, na porção nordeste do estado, Grão Mogol, Araçuaí, Peçanha e Almenara apresentaram IDH abaixo de 0,611.^11^

Todos os óbitos por AVC de Minas Gerais, no período avaliado, foram classificados pelas seguintes variáveis:

sexo (homens; mulheres);
idade (em anos: ≤19, 20-39, 40-49, 50-59, 60-69, 70-79, ≥80);
local de residência do óbito (microrregião);
ano do óbito (1980–2021).

No período de estudo, do total de 392.521 óbitos, 441 (1,1%) não tinham a informação da microrregião de residência, portanto não foram utilizados na análise espacial.

Os dados de mortalidade foram extraídos do Sistema de Informação sobre Mortalidade (SIM) do Ministério da Saúde,^2^ de acordo com a microrregião de residência dos óbitos registrados. Para o período de 1996 a 2021, as mortes por AVC correspondem aos códigos I60 a I69, previstos na Décima Revisão da Classificação Estatística Internacional de Doenças e Problemas Relacionados à Saúde (CID-10). Para o período de 1980 a 1995, as mortes por AVC correspondem aos códigos 430 a 438 da CID-9.^2^ Dados populacionais por faixa etária, sexo e período foram obtidos dos censos demográficos, com as respectivas projeções para os períodos intercensitários, disponíveis no portal do Departamento de Informação e Informática do Sistema Único de Saúde (DATASUS).^2^ A base cartográfica do estudo constituiu-se da malha territorial do estado de Minas Gerais, por microrregiões, no formato *shapefile*, disponível no portal do IBGE.^10^

Para a análise das tendências das taxas de mortalidade para o estado de Minas Gerais, foi utilizado o Joinpoint Regression Program, versão 5.0.2.^12^ Foram calculadas as taxas de mortalidade bruta, ajustadas por idade e específicas por faixa etária. O cálculo da taxa de mortalidade bruta partiu do número de óbitos por AVC de residentes em determinado local, considerado como numerador, e da população total residente, como denominador, multiplicado por 100 mil, no período considerado. A taxa específica por faixa etária levou em conta o número de óbitos de uma faixa etária dividido pela população da mesma faixa. A taxa de mortalidade ajustada por idade foi calculada pelo método direto, com a população padrão da Organização Mundial da Saúde (OMS).^13^ Essa abordagem ajusta as taxas brutas de acordo com a distribuição etária de uma população externa, arbitrariamente definida (neste caso, a população padrão da OMS), o que é útil para comparar populações de diferentes estruturas etárias.

O Joinpoint Regression Program utiliza o modelo de regressão log-linear de Poisson segmentada, que aplica o teste de permutação de Monte Carlo para identificar pontos em que a linha de tendência muda significativamente em magnitude ou direção. A análise começa com o número mínimo de *joinpoints* (zero, que é uma linha reta), e testa se um ou mais *joinpoints* são estatisticamente significativos e devem ser adicionados ao modelo. A VPA e o intervalo de confiança de 95% (IC_95_%) foram estimados para os segmentos de tempo em ambos os lados dos pontos de inflexão. Para este estudo, os parâmetros selecionados foram: método *grid search* (os pontos de junção ocorrem exatamente nas observações), duas observações mínimas entre pontos de junção e no máximo cinco pontos de junção por análise. O programa Joinpoint tem sido utilizado na área da epidemiologia para estimar as tendências da mortalidade por diversas causas, incluindo-se o AVC.^4,14-16^

Para a descrição da evolução do padrão espacial das taxas de mortalidade, foram elaborados mapas temáticos coropléticos.^17^ Foram utilizadas as taxas ajustadas por sexo e idade, agrupadas em quinquênios, com exceção do último período (sete anos): 1980 a 1984, 1985 a 1989, 1990 a 1994, 1995 a 1999, 2000 a 2004, 2005 a 2009, 2010 a 2014, e 2015 a 2021. O método usado para definir os intervalos das legendas foi o de Jenks.

A identificação dos conglomerados espaço-temporais foi conduzida pela estatística *scan*, com o programa SaTScan versão 10.1.3.^18^ Essa estatística insere uma janela circular de tamanho variável no centroide de cada área, de maneira que o raio aumente de tamanho, varrendo e adicionando os centroides das áreas vizinhas. O tamanho do conglomerado, ou janela espacial, não pode ultrapassar 50% da população. Sob a hipótese nula, calcula-se o risco relativo (RR) do conglomerado e verifica-se se o mesmo ocorreu ao acaso, utilizando-se para tanto o teste da razão de verossimilhança com base nas simulações de Monte Carlo. Nesta análise, as variáveis “faixa etária” e “sexo” foram consideradas como variáveis de ajuste, de tipo *offset*. Foram conduzidos quatro tipos de análise para identificação de conglomerados no SaTScan: i) Puramente espacial, ii) espaço-temporal para taxas altas, iii) espaço-temporal para taxas baixas e iv) variação espacial nas tendências temporais. O SaTScan considera o conglomerado de taxas altas quando o RR é significativamente maior do que 1, e de taxas baixas quando o RR é inferior a 1. A análise espaço-temporal identifica em qual período os RR dos respectivos conglomerados foram significativos. A janela temporal não pode ultrapassar 50% do período de estudo. A análise de variação espacial nas tendências temporais identifica a VPA das taxas dentro e fora dos conglomerados. O nível de significância adotado foi de 5% para todos os testes. Para elaboração dos mapas, foi utilizado o programa ArcGIS versão 10.6.

De acordo com a Resolução nº 466/2012 do Conselho Nacional de Saúde, o estudo utilizou dados secundários de domínio público do DATASUS e, portanto, não necessitou ser submetido à apreciação de Comitê de Ética em Pesquisa. 

## RESULTADOS

No período estudado, ocorreram 392.521 mortes por AVC, o que corresponde a uma taxa de mortalidade de 52,6/100 mil habitantes-ano. A Figura 2 apresenta as tendências das taxas de mortalidade por AVC em Minas Gerais, de 1980 a 2021. Os pontos representam os valores observados, e as linhas correspondem às tendências da análise de regressão. Tanto a taxa bruta quanto a ajustada apresentaram tendência de diminuição significativa. A velocidade foi maior na taxa ajustada, sobretudo a partir de 1994, com VPA de -3,69 até o final do período (Figura 2A).

**Figura 2:** Tendências das taxas de mortalidade por acidente vascular cerebral (por 100 mil), em Minas Gerais, de 1980 a 2021: A) Taxa ajustada e taxa bruta, B) Até 19 anos e de 20 a 39 anos, C) De 40 a 49 e de 50 a 59, D) De 60 a 69 anos, de 70 a 79 anos, de 80 anos e mais

As faixas etárias mais jovens apresentaram as menores taxas de mortalidade. A análise de tendência revelou que todas as faixas etárias apresentaram tendência de diminuição significativa em todo o período, ou seja, zero *joinpoint*. A faixa etária de 20 a 39 anos apresentou a maior velocidade de diminuição (VPA = -4,48), e a faixa etária até 19 anos, a menor (VPA = -1,74) (Figura 2B). 

A Figura 3 apresenta a evolução espaço-temporal das taxas de mortalidade por AVC. Nota-se que os primeiros quinquênios apresentaram as maiores amplitudes de variação das taxas e também a redução das taxas no decorrer do período. No primeiro quinquênio, a taxa média foi de 98,6/100 mil habitantes. Nota-se maiores taxas, acima de 150/100 mil, nas porções sudeste e sul do estado, nas microrregiões de Cataguases, Barbacena e Juiz de Fora. Em contrapartida, Grão Mogol, Januária e Araçuaí, ao norte, apresentaram taxas inferiores a 35/100 mil habitantes (Figura 3A).

**Figura 3:** Taxas de mortalidade por acidente vascular cerebral (por 100 mil) ajustadas por sexo e idade, em Minas Gerais, de 1980 a 2021, por quinquênios (com exceção do último período de sete anos)

No segundo quinquênio, o padrão espacial foi semelhante (Figura 3B). Destacaram-se também as microrregiões da região metropolitana, como Belo Horizonte, Ouro Preto, Pará de Minas, Itabira, Conselheiro Lafaiete, com taxas acima da média. No terceiro e quarto quinquênios, as taxas médias foram de 85,2/100 mil habitantes e 66,7/100 mil habitantes, respectivamente. O padrão espacial se manteve, com maiores taxas na porção sul e sudeste (Figura 3C e D). No quinto e sexto quinquênios, as taxas médias foram de 57,7/100 mil habitantes e 55,4/100 mil habitantes, respectivamente (Figura 3E e F).

No sétimo quinquênio, a taxa média do estado foi de 46,8/100 mil habitantes. Um novo padrão começa a se formar, com taxas altas em microrregiões do norte (Bocaiúva) e do leste (Governador Valadares) (Figura 3F). No último quinquênio, a taxa média foi de 36,8/100 mil habitantes, a menor em todo o período abarcado pelo estudo (Figura 3H). O sul perde o protagonismo; destacaram-se na porção leste as microrregiões de Nanuque, Teófilo Otoni e Governador Valadares, com taxas altas. 

A Figura 4 apresenta o resultado do teste de varredura espacial. Na análise puramente espacial, foram detectados dois conglomerados, um dos quais de taxas altas, no sul, composto por 22 áreas, incluindo 75% das microrregiões no sul da região metropolitana (Belo Horizonte, Conselheiro Lafaiete, Itabira, Itaguara, Ouro Preto, Pará de Minas). Aí, o risco relativo foi de 1,20; portanto, os residentes nesse conglomerado possuem o risco de morrer por AVC aumentado em 20% em relação às áreas fora do conglomerado (Figura 4A). Em contrapartida, ao norte foi identificado um conglomerado de taxas baixas, formado por 21 áreas. O risco de morrer por AVC nessa área é diminuído em 24% (Figura 4A). 

**Figura 4:** Teste de varredura espacial da mortalidade por acidente vascular cerebral no estado de Minas Gerais, de 1980 a 2021: A) Análise puramente espacial, B) Análise espaço-temporal para taxas altas, C) Análise espaço-temporal para taxas baixas, D) Análise de variação espacial nas tendências temporais

A análise espaço-temporal para taxas altas identificou um conglomerado no período de 1980 a 1999 (Figura 4B), formado pelas mesmas 22 microrregiões da análise puramente espacial (Figura 4A). No entanto, nesse conglomerado, de 1980 a 1999, o risco de morte por AVC é duas vezes maior. A análise espaço-temporal para taxas baixas identificou um conglomerado no período de 2008 a 2021, formado por 28 áreas a noroeste e oeste do estado (Figura 4D). Nessa área, o risco de morte por AVC é diminuído em 41%. Esse conglomerado incluiu a microrregião de Belo Horizonte e a porção oeste da região metropolitana. Por fim, a análise de variação espacial nas tendências temporais revelou que a tendência de diminuição foi maior no conglomerado de taxas altas (VPA = -3,64) (Figura 4C).

## DISCUSSÃO

A mortalidade por AVC em Minas Gerais tem apresentado tendência de diminuição na taxa bruta, na taxa ajustada por idade e em todas as faixas etárias. Um estudo avaliou as tendências das taxas de mortalidade por AVC no mundo, de 1990 a 2019, com dados do projeto Carga Global de Doenças (Global Burden of Disease).^3^ A taxa global de mortes por AVC indicou tendência de diminuição, com VPA = -1,58. O estudo criou um índice sociodemográfico sintético, composto pelas seguintes variáveis: renda *per capita*, escolaridade média e taxa de fertilidade total. O índice foi categorizado em cinco quintis: alto, médio-alto, médio, médio-baixo e baixo. Países com alto índice sociodemográfico apresentaram maior velocidade de redução (VPA = -2,83), que diminuiu gradativamente, passando pelo índice médio, que inclui o Brasil (VPA = -1,37), até os países com baixo índice sociodemográfico (VPA = -1,21). 

As tendências das taxas de mortalidade por AVC foram estudadas no Brasil, por sexo e faixa etária, de 2000 a 2018.^16^ O estudo descreveu diminuição em todas as faixas etárias, sendo mais intensa na faixa de 35 a 44 anos, com redução de 52,1% em homens e 53,2% em mulheres. Porém, não relatou as VPAs e respectivas significâncias estatísticas, apresentando os resultados em gráficos. Minas Gerais parece seguir o padrão nacional, dado que a velocidade de diminuição foi mais intensa nas faixas etárias de 20 a 49 anos.

As tendências das mortes por AVC também foram analisadas no Brasil por estado e região, de 1996 a 2015.^4^ Para o Brasil, a VPA foi de -2,4. Todos os estados das regiões Sul, Sudeste e Centro-Oeste apresentaram tendência de diminuição no período, que foi mais intensa no Sul e no Sudeste. No Nordeste, mais da metade dos municípios apresentaram tendência de aumento. No Norte, o predomínio foi de estabilidade. Apesar do declínio da mortalidade por AVC no Brasil, houve crescimento da mortalidade por hipertensão, possivelmente relacionado ao preenchimento das declarações de óbito, com o declínio das causas mal definidas e aumento de hipertensão.^19^ Novamente, Minas Gerais parece seguir o padrão nacional, dado que a velocidade de redução nas taxas foi maior na porção sul do estado.

Em parte, esse padrão espaço-temporal das mortes por AVC pode ser explicado pelo processo de transição epidemiológica. No Brasil, houve o atraso de algumas décadas em relação aos países ricos. Em países como Estados Unidos, Canadá e nações da Europa ocidental, a mortalidade por doença cardiovascular iniciou o declínio no final da década de 1950.^20^ A principal mudança no padrão nacional ocorreu na década de 1960, quando a mortalidade por doenças cardiovasculares superou as doenças infecciosas.^21^ A mortalidade por doença cardiovascular continuou a crescer, e começou a diminuir a partir da década de 1980.^20,22^ Além disso, no Brasil, a transição ocorre de forma prolongada, com a tênue redução e reintrodução de doenças infecciosas, como a dengue. Adicionalmente, há diferenças regionais, com atraso do Norte e do Nordeste em relação ao Sul e ao Sudeste.^20^

Diversos fatores estão relacionados ao processo de transição epidemiológica, como o acesso aos serviços de saúde,^20^ o aumento da expectativa de vida e da taxa de urbanização,^21^ a melhora das condições sanitárias e nutricionais, o aumento da cobertura vacinal e as mudanças dos hábitos de vida, como a redução do tabagismo.^23,24^ Em Minas Gerais, informações sobre causa de morte por macrorregiões de saúde, levantadas pelo SIM,^2^ sugerem a transição prolongada e o atraso no norte em relação ao sul. No sul, as mortes por doenças cardiovasculares diminuíram de 33,1%, em 1980, para 26,7% em 2019. Na macrorregião norte, em período equivalente, o valor aumentou de 14,9% para 21,6%. No caso das doenças infecciosas, na macrorregião sul, o percentual diminuiu de 7,5%, em 1980, para 2,5% em 2019. Na macrorregião norte, o valor diminuiu de 11,4% para 6,2%.

No Brasil, estudos sobre a evolução espaço-temporal da mortalidade por AVC com informações intraestaduais são escassos. A evolução espaço-temporal da mortalidade por AVC foi analisada na população idosa em Alagoas, de 2000 a 2016, por meio da análise de tendência e pela estatística de Moran.^15^ O estudo identificou tendência de aumento até 2007 (VPA = +3,9) e diminuição até 2016 (VPA = -1,4). Quanto ao padrão espacial, identificou um conglomerado do tipo alto-alto a leste, incluindo a capital Maceió, e outro do tipo baixo-baixo a oeste do estado.^15^ A tendência de queda a partir de 2007, bem como a área de risco, que inclui a capital, pode ser uma evidência do atraso no processo de transição epidemiológica. 

A evolução espaço-temporal da mortalidade por AVC foi estudada no estado do Rio de Janeiro, por meio de agregação dos municípios em regiões, com exceção da capital e de Niterói,^25^ em três períodos: 1979 a 1989, 1990 a 1999, e 2000 a 2010. No primeiro período, as taxas foram maiores nas regiões serrana e noroeste, e menores ao sul, em municípios que incluíram a capital e Niterói. Todas as regiões apresentaram diminuição na mortalidade por AVC, sendo esta maior nas regiões serrana e noroeste. A capital e Niterói mantiveram taxas baixas nos períodos subsequentes, sendo que, no terceiro período, houve homogeneização em todo o estado. 

Outros estudos investigaram a evolução espaço-temporal da mortalidade por doenças cardiovasculares, que incluem o AVC.^4,9,26,27^ No Ceará, um estudo analisou espacialmente a mortalidade por doenças cardiovasculares, entre 2009 e 2019, por meio do índice de Moran local.^9^ Foi identificado um conglomerado do tipo alto-alto no sul cearense, e um do tipo baixo-baixo no litoral norte e na Região Metropolitana de Fortaleza. No Ceará, também foi identificada tendência de aumento nas taxas de mortalidade por AVC de 1996 a 2007 (VPA = +2,8) e de diminuição de 2007 a 2015 (VPA = -2,2).^4^ Essas informações também sugerem o processo de atraso na transição epidemiológica no Nordeste. 

No estado do Paraná, um estudo investigou a distribuição espacial da mortalidade por doenças cardiovasculares por macrorregiões de saúde, nos triênios 1989-1991 e 2006-2008.^26^ No primeiro triênio, a macrorregião norte apresentou maior taxa, e a oeste, a menor. No segundo triênio, a macrorregião noroeste apresentou o maior risco, e a leste, que inclui a capital, o menor. No entanto, o estudo não detectou dependência espacial pelo índice global de Moran. O padrão espacial de diversas causas de morte, incluindo doenças cardiovasculares, foi analisado no estado de São Paulo, em 2000, 2008 e 2016.^27^ O estudo identificou maior concentração de mortes no sudoeste e noroeste nos três anos, sendo que, em 2016, foram identificados alguns *hotspots* ao norte do estado. 

O presente estudo possui algumas limitações. Uma delas são eventuais falhas no preenchimento das declarações de óbito, como possíveis erros relacionados à CID-10 e à CID-9, que pode variar em diferentes regiões.^19,20^ Sabe-se que houve melhora na qualidade da informação referente ao SIM, nas últimas décadas. A proporção de óbitos por causas mal definidas (Capítulo XVIII da CID-10)^28^ em Minas Gerais, em 1996, foi de 14,4%, abaixo da média nacional (15,1%) e da região Nordeste (32,4%), porém acima da região Sudeste (9,2%). Por sua vez, em 2021, houve melhora, e a proporção diminuiu de forma geral: Minas Gerais (5,9%), média nacional (5,1%), e regiões Sudeste (5,7%) e Nordeste (5,9%).^2^ Outra limitação é inerente ao desenho do estudo. Trata-se da falácia ecológica, ou seja, atribuir ao indivíduo resultado encontrado em agregados populacionais. A unidade de análise adotada, a microrregião, também pode ocultar desigualdades na escala municipal. A incompletude dos dados (1,1%) não interferiu nos resultados.

Conclusivamente, o presente estudo identificou, em Minas Gerais, tendência geral de diminuição na mortalidade por AVC. A identificação de conglomerados, bem como a constatação de taxas altas nos últimos anos no nordeste do estado, podem auxiliar os gestores na formulação de políticas públicas para redução dos riscos e da mortalidade por AVC, promovendo-se melhor qualidade de vida para a população.
